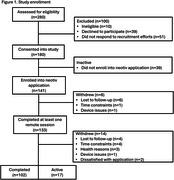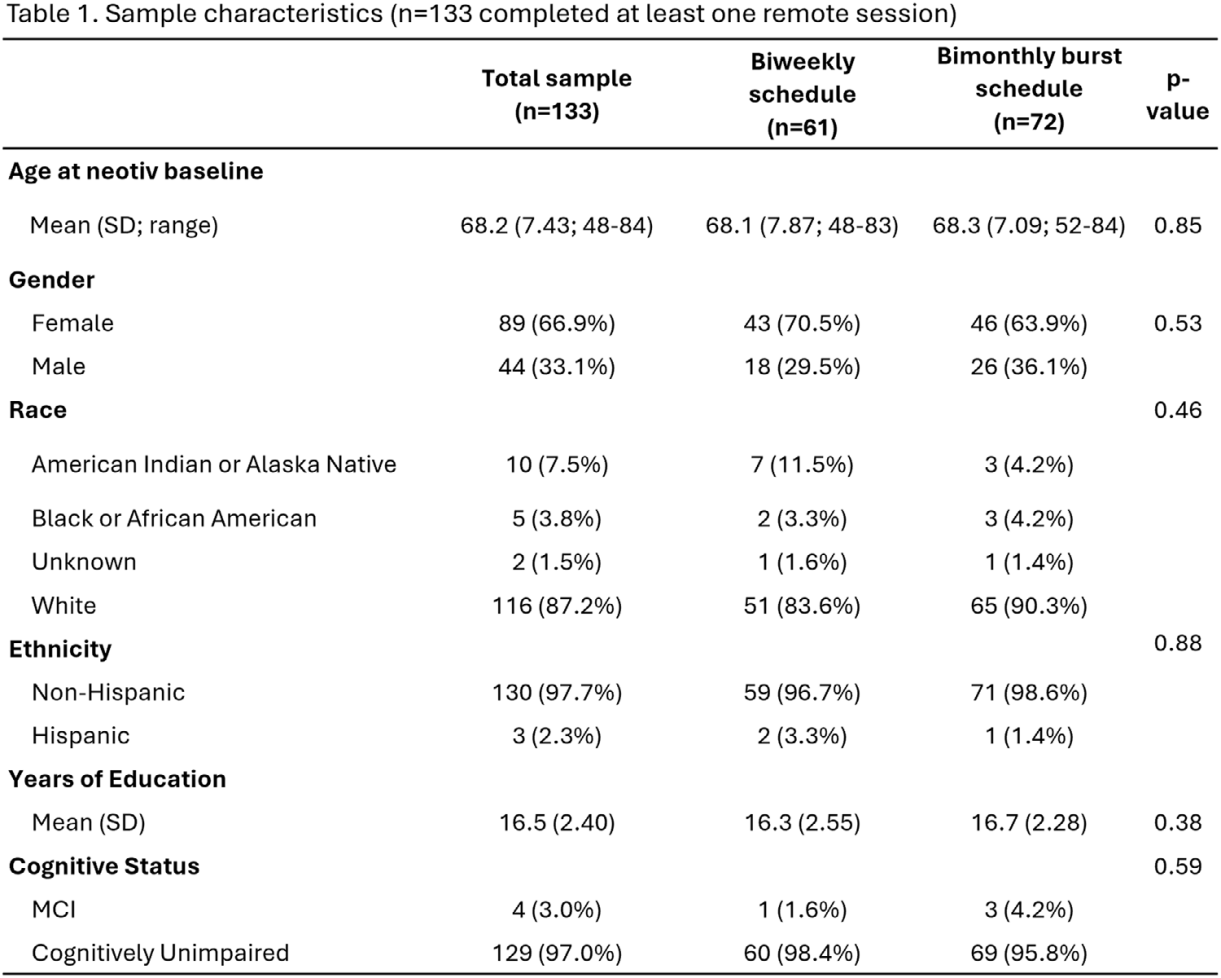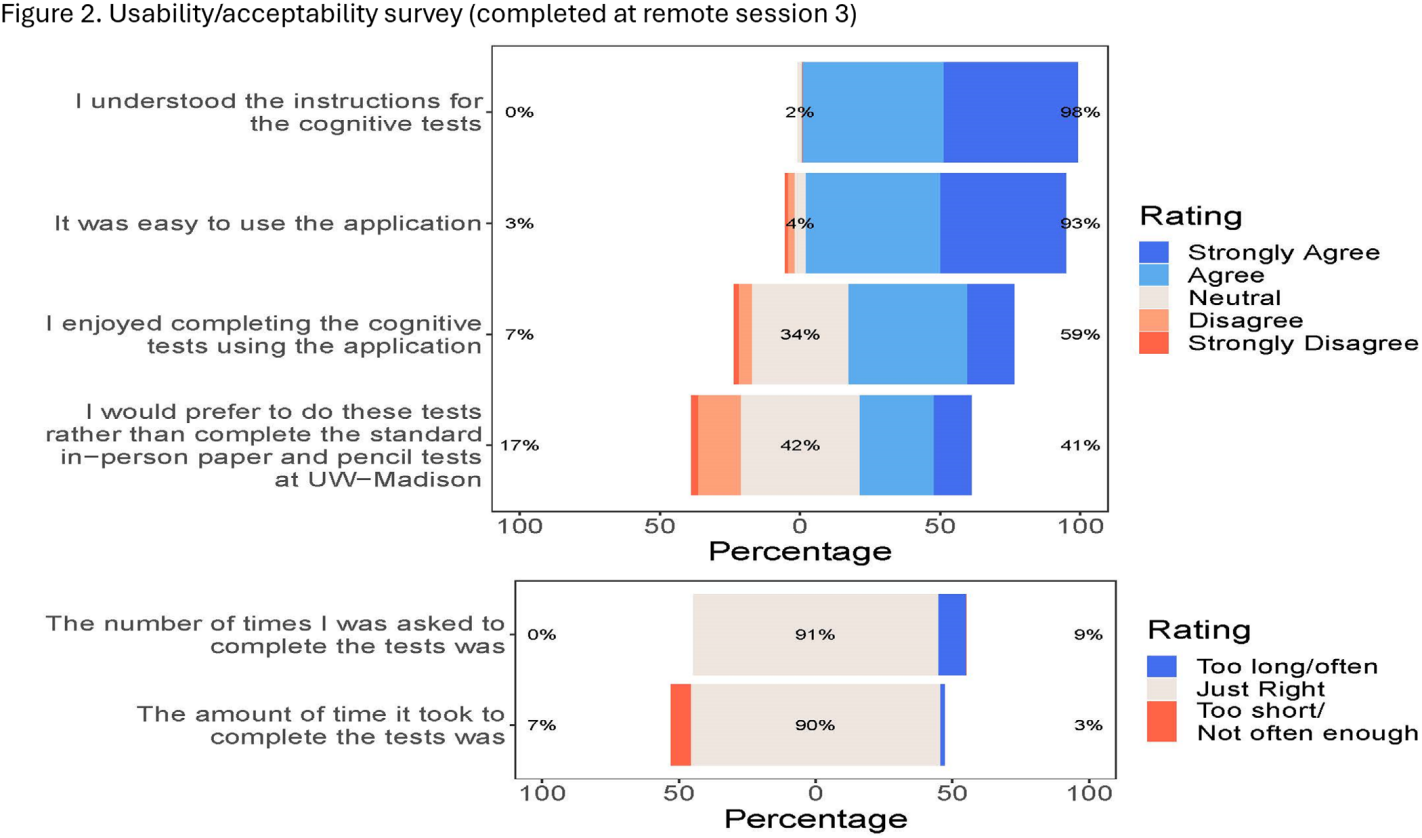# Remote digital memory assessment in middle‐aged and older adults: Feasibility, reliability, and construct validity

**DOI:** 10.1002/alz.089786

**Published:** 2025-01-03

**Authors:** Lindsay R. Clark, Kristin E Basche, Amanda Peterson, Hannah Rosario, Sterling C. Johnson, Emrah Düzel, David Berron

**Affiliations:** ^1^ University of Wisconsin‐Madison School of Medicine and Public Health, Madison, WI USA; ^2^ William S. Middleton Memorial Veterans Hospital, Madison, WI USA; ^3^ University of Wisconsin School of Medicine and Public Health, Madison, WI USA; ^4^ German Center for Neurodegenerative Diseases (DZNE), Magdeburg Germany

## Abstract

**Background:**

Frequent and remote cognitive assessment may improve sensitivity to subtle cognitive decline associated with preclinical Alzheimer’s disease (AD). Our objective was to evaluate the feasibility, reliability, and construct validity of repeated remote memory assessment in late middle‐aged and older adults.

**Method:**

Participants were recruited from longitudinal aging cohorts to complete medial temporal lobe‐based memory paradigms (Object‐In‐Room Recall [ORR], Mnemonic Discrimination for Objects and Scenes [MDT‐OS], Complex Scene Recognition [CSR]) using the neotiv application on a smartphone or tablet at repeated intervals over one year. Participants were randomized to a task schedule (biweekly or bimonthly burst) for a total of 24 ten‐minute remote sessions. Feasibility metrics included participation, retention, compliance, and acceptability. Intraclass correlation coefficients (ICC) assessed test‐retest reliability over an 8‐week period. Analysis of covariance (ANCOVA) models including covariates of demographics, device type, and task schedule were used to evaluate relationships between neotiv baseline performance and traditional memory scores (Rey Auditory Verbal Learning Test [RAVLT]; Preclinical Alzheimer’s Cognitive Composite [PACC]).

**Result:**

Of 280 potentially eligible adults, *n* = 180 consented (64% participation rate). Of 180 consented, *n* = 133 completed at least one session and *n* = 119 completed or nearly completed all sessions (retention rate = 66% for all consented and 89% for all completed 1+ session). Delayed retrieval sessions were completed within expected timeframes in 72% of sessions. Greater than 90% of respondents felt the application was easy to use, instructions were understood, and the frequency and length of remote sessions were appropriate. 60% enjoyed completing the tasks (34% felt neutral) and 40% preferred mobile tests to traditional cognitive testing (42% felt neutral). Test‐retest reliability was moderate (ICC = 0.54‐0.70). RAVLT and PACC were significantly associated with MDT‐OS, CSR, and ORR performance (*p*’s <.001).

**Conclusion:**

Challenges to participation occurred primarily during enrollment; future studies may allocate additional support facilitating registration and initial task completion within the application. Acceptability of the mobile tasks was high. The mobile memory paradigms exhibited moderate test‐retest reliability and were significantly related to gold‐standard cognitive tests. Overall, findings support preliminary feasibility, reliability, and validity of the neotiv tasks in assessing memory function in cognitively healthy adults enriched for AD risk.